# Genetic structure and *kdr* mutations in *Aedes aegypti* populations along a road crossing the Amazon Forest in Amapá State, Brazil

**DOI:** 10.1038/s41598-023-44430-x

**Published:** 2023-10-11

**Authors:** Barbara S. Souza, Leticia F. Lima, Allan K. R. Galardo, Vincent Corbel, Jose Bento P. Lima, Ademir J. Martins

**Affiliations:** 1 Laboratório de Biologia, Controle e Vigilância de Insetos Vetores (LBCVIV), Instituto Oswaldo Cruz (IOC)/FIOCRUZ, Rio de Janeiro, RJ 21040-900 Brazil; 2 Laboratório de Bioinformática, Instituto de Química (UFRJ), Rio de Janeiro, RJ 21941-909 Brazil; 3 Instituto de Pesquisas Científicas e Tecnológicas do Estado do Amapá/IEPA, Macapá, AP 68.908-220 Brazil; 4 Institut de Recherche Pour le Développement (IRD), MIVEGEC, CNRS, IRD, Université de Montpellier, 34090 Montpellier, France; 5 Instituto Nacional de Ciência e Tecnologia em Entomologia Molecular (INCT-EM), UFRJ, Rio de Janeiro, RJ 21941-902 Brazil

**Keywords:** Population genetics, Entomology, Genetic markers

## Abstract

Insecticide resistance in *Aedes aegypti* poses a significant threat to disease control. One form of resistance, caused by *kdr* mutations in the *Na*_*V*_ gene, hinders vector control efforts in Brazil. Despite genetic differences typically accumulating among isolated populations, this mosquito can actively and passively disperse through human transportation. Our study investigated the genetic structure and spread of *kdr* mutations in *Ae. aegypti* populations across six localities in Amapá State, Brazil, within the Amazonian Forest. Using 12 microsatellite loci and qPCR methods, we assessed genetic structure and identified three common *kdr* mutations (V410L, V1016I, and F1534C). High prevalence of *kdr* alleles was observed in all localities, indicating widespread distribution in Amapá State. Microsatellite analysis revealed differentiation among mosquito populations, dividing them into two distinct clusters supported by Bayesian and DAPC analyses. Oiapoque, located along the northern border with French Guiana, exhibited the highest *kdr* frequencies and genetic differentiation compared to other localities. Our findings suggest genetic structure in *Ae. aegypti* populations in Amapá State, with some passive gene flow between clusters. The study underscores the need for continuous surveillance of *Ae. aegypti* populations to monitor the spread of insecticide resistance and inform effective vector control strategies.

## Introduction

Arboviruses (*Arthropod-borne virus*) infections, such as dengue, Zika, chikungunya and yellow fever are causing severe impacts on global health, especially in tropical low-income countries^1^. In Brazil, dengue is considered as hyperendemic, with regional and seasonal circulation of the four serotypes (I–IV), but other newly emerged viruses such as Zika (ZIKV) and chikungunya (CHIKV) are also circulating^2^. In 2022, Brazil reached the highest number of deaths caused by dengue fever (1016 deaths) ever recorded in the country^3^, and the recent increase in yellow fever cases in regions bordering urban centers^4^ highlights the imminence of re-urbanization of this disease^5^. As there are no effective vaccines available for most of these arboviral diseases (excluding yellow fever), controlling the vector—the mosquito *Aedes aegypti* (Diptera, Culicidae: Linnaeus, 1762)—is the method of choice for reducing the risk of arbovirus transmission.

Chemical control is still the cornerstone of any vector borne-diseases control programme worldwide^6,7^. Historically, insecticides have played an essential role in the decline of various diseases, including malaria and dengue^8^. However, the massive and repeated use of chemicals for decades has favored the spread of insecticide-resistance in *Aedes* mosquitoes^9^. Currently, insecticide resistance (IR) is seen as one of the most important threats for the control of diseases caused by viruses transmitted by *Aedes,* as it may reduce the efficacy of chemical-based vector control interventions^10,11^. In Brazil, nationwide insecticide resistance monitoring (MRI) has reported strong resistance of *Ae. aegypti* to all pesticides used by the Ministry of Health, such as the larvicide temephos (organophosphate), the insect growth regulator pyriproxyfen, and the adulticides deltamethrin (pyrethroid) and malathion (organophosphate)^12,13^.

Among the genetic alterations involved in IR, the most common are single nucleotide changes in the voltage-gated sodium channel (*Na*_*V*_), which cause resistance to the knockdown effect of pyrethroids, and are therefore called the *kdr* mutations (knockdown resistance)^14^. In Brazil, at least three *kdr* mutations have been reported in *Ae. aegypti*: a substitution of Valine to Isoleucine at position 1016 (V1016I), a Phenylalanine to Cysteine at position 1534 (F1534C), and a Valine to Leucine at position 410 (V410L)^15,16^. These mutations were classified as *Na*_*V*_*R1* and *Na*_*V*_*R2*, where *Na*_*V*_*R1* is a haplotype containing the F1534C *kdr* mutation only while *Na*_*V*_*R2* exhibits F1534C + V410L + V1016I mutations^17^. These alleles give pyrethroid resistance under a recessive trait, and the *Na*_*V*_*R2* causes the highest level of resistance to deltamethrin^18^. Other SNPs, such as V1016G, S989P and T1520I, are common in Asian populations of *Ae. aegypti*^19,20^ but they are absent in Brazil and neighboring countries.

Monitoring the presence and spread of *kdr* mutations through molecular methods is relevant because pyrethroids are widely used for vector control and household domestic purposes^21,22,16^. Additionally, the analysis of the genetic structure and gene flow among vector populations can contribute to a better understanding of the evolutionary forces driving the dispersion of resistance alleles^23^.

In Brazil, differences in the genetic structure and spatial distribution of pyrethroid (*kdr*) resistance have been reported in *Aedes aegypti* with at least three well defined clustered regions within the country^16^. The Amapá State (AP), located in the Northern part of the country, bordering the French Guiana, is an important gateway between Brazil and the Caribbean to arboviruses and vector populations with distinct characteristics, including insecticide resistance^24^. By the way, this was one of the entrance doors for the CHIKV virus in 2015 in Brazil^25^. In addition, the geographical aspects of the region raise relevant questions for population genetic studies since locations infested with *Ae. aegypti* are relatively isolated from each other’s due to the vast Amazonian vegetation. In AP, *Ae. aegypti* from Oiapoque (the city bordering the French Guiana) is among the Brazilian mosquito populations exhibiting the highest levels of resistance to deltamethrin, temephos and malathion, while the *Ae. aegypti* population from the AP State capital, Macapá city, is far less resistant^12,26,27^.These cities are 577 km distant, connected by a road (BR-156) that crosses the dense Amazon Forest. The frequency of *kdr* alleles is distinct between them, with a predominance of *kdr Na*_*V*_*R1* and *kdr Na*_*V*_*R2* in Macapá and Oiapoque, respectively^24^. The presence and frequency of these *kdr* alleles in other cities of the AP State is however unknown.

In this study, we conducted mosquito collection in cities located along the BR-156 road of the Amapá State to assess the genetic structure and the spatial distribution of *kdr* mutations in *Ae. aegypti* along a South-North transect. The study was carried out to better understand local adaptation of dengue vector populations in the Amapá State and guide national authority in the selection of the most judicious insecticides to use for vector control.

## Results

### *Kdr* genotyping

**Figure 1:**
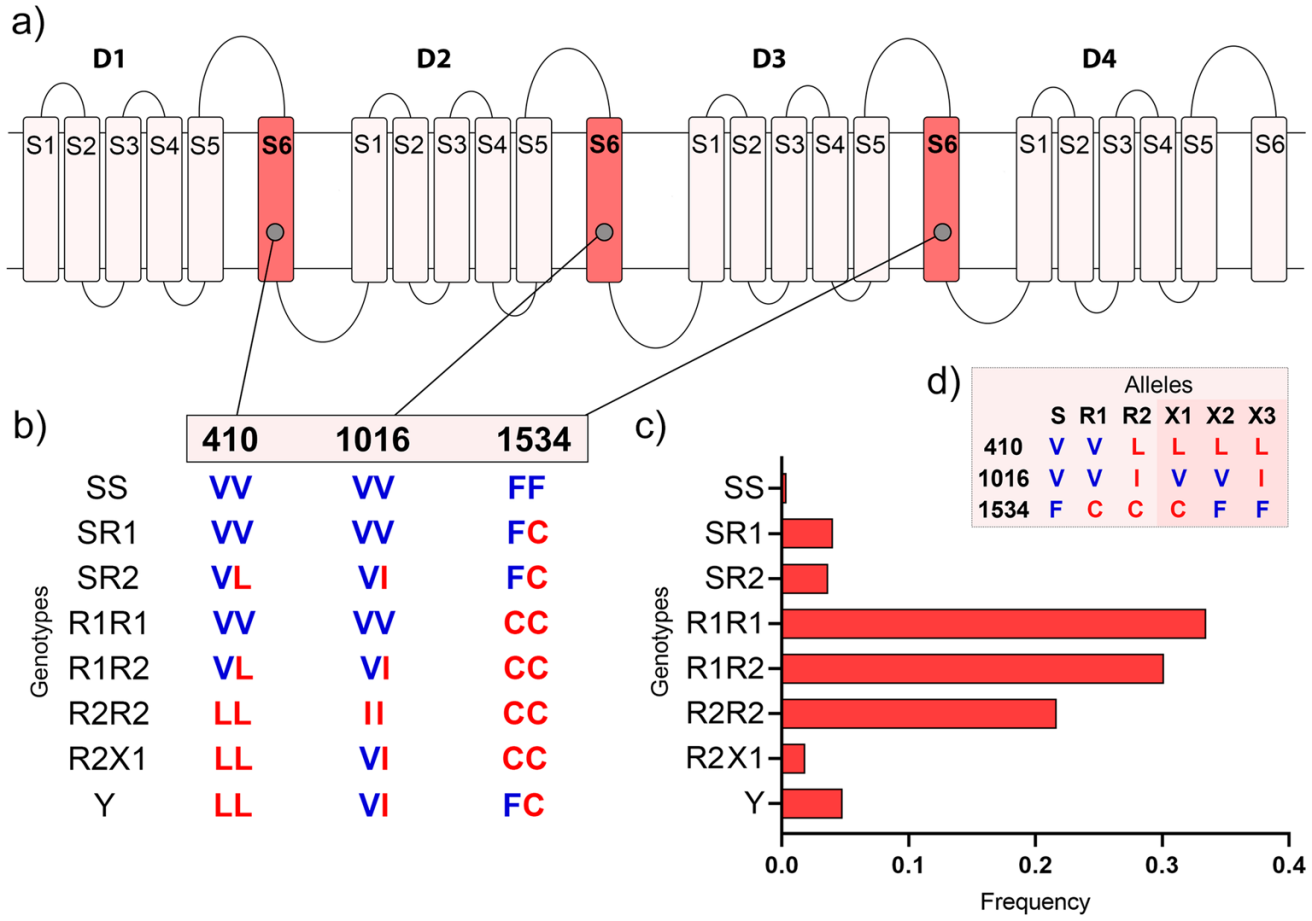
*Kdr* genotypes in *Aedes aegypti* from the Amapá State, Brazil. (**a**) Scheme of the voltage-gated sodium channel (*Na*_*V*_), indicating the four domains (D1–D4), each with six transmembrane segments (S1–S6); (**b**) The genotypes observed in each of the three SNP sites in IS6, IIS6 and IIIS6 *Na*_*V*_ segments. The wild-type and the *kdr* genotypes are in blue and red, respectively; (**c**) The total *kdr* genotypic frequencies, considering all samples (272 mosquitos genotyped for V410L, V1016I and F1534C) from the Amapá State; (**d**) Possible alleles that constitute the genotypes found here. The "Y" genotype is obtained by combining R2/X2 or X1/X3 alleles.

We genotyped 272 *Ae. aegypti* mosquitoes for the three *kdr* SNPs V410L, V1016I and F1534C. In total, we found eight genotypes (Fig. 1): the six genotypes expected by the combination of wild-type *Na*_*V*_*S* (VVF), *kdr Na*_*V*_*R1* (VVC) and *kdr Na*_*V*_*R2* (LIC) alleles, and two additional. The genotype R2X1 (LL + VI + CC), composed of *kdr Na*_*V*_*R2* and the herein called *kdr Na*_*V*_*X1* (LVC) alleles, was found in OIA and CAL. We could not accurately determine the allelic composition of the genotype Y (LL + VI + FC), because it may be composed of LIC/LVF or LVC/LIF. This genotype was exclusively found in OIA. We named the possible alleles LFV and LIF as *kdr Na*_*V*_*X2* and *kdr Na*_*V*_*X3*, respectively (Supplementary Tables S1, S2 and Fig. 1).

In general, the lowest genotype frequency found in the *Ae. aegypti* populations was the wild-type homozygous SS (VV + VV + FF), only observed in MAC (2.3%). The most frequent genotype was R1R1 (VV + VV + CC), ranging from 2.2% in OIA to 65.1% in MAC. All populations showed high frequencies of other “resistant genotypes” (i.e. R1R1, R1R2, and R2R2), whereas their sum ranged from 63% in OIA to 100% in PGR. MAC was the only population without the homozygous R2R2 genotype (R1R1 + R1R2 = 76.7%). The genotype R2X1 was observed in CAL (8.7%) and OIA (2.2%). The genotype Y (LL + VI + FC) was found only in OIA, although at a relatively low frequency (28.3%) (Supplementary Table S1 and Fig. 1). The populations showed opposite trends regarding the *kdr* allelic frequencies according to their location along the South-North transect. For example, MAC presented the higher *kdr Na*_*V*_*R1* (77.9%) and the lower *kdr Na*_*V*_*R2* (9.3%) frequencies, while OIA showed the lower *kdr Na*_*V*_*R1* (15.2%) and the higher *kdr Na*_*V*_*R2* (52.2%) frequencies (Supplementary Table S2). The genotypic frequencies of each population are presented in Supplementary Figure S1.

### *Kdr* haplotype sequences

We amplified and sequenced the corresponding IS6, IIS6 and IIIS6 *Na*_*V*_ fragments of some homozygous R1R1 (4 MAC and 1 OIA) and R2R2 (4 OIA) mosquitoes. The IS6, IIS6 and IIIS6 sequences of R1R1 samples were similar to previously published sequences (GenBank accession numbers: LC557528, MN602762, MN602780, respectively). The R2R2 sequences of samples from OIA, IS6, IIS6, and IIIS6 fragments were also similar to known sequences (GenBank accession numbers: KY747530, MN602754, MN602780, respectively).

### Microsatellite analyses

We genotyped 12 microsatellite loci in a total of 288 *Ae. aegypti* divided into six populations from the Amapá State. We observed a total of 60 alleles, varying from two (AC4 and B2) to 17 (Ag2) (Supplementary Table S3). Some markers were not under HWE, even after Bonferroni corrections, in some populations: AG2 (CAL, OAI), A9 (TTZ, CAL), A1 (FGO), AC1 (FGO, TTZ), AG5 (FGO, CAL) and AG1 (FGO) (Supplementary Table S4). In this case, the populations showed a Fis > 0 (Table 1 and Supplementary Table S4), indicating a heterozygous deficit. Although the LD test showed 33 tests significant out of the 396 evaluated combinations (8.3%), none loci pairs were consistently correlated in all populations after Bonferroni correction (Supplementary Table S5).

**Table 1:** Genetic diversity of six *Aedes aegypti* populations from Amapá State, Brazil, based on the analysis of 12 microsatellites.

Pop	N	Na	Ne	Ho	He	Np	R	Fis
CAL	47.917	3.583	2.497	0.528	0.559	0.000	3750	0.052
OIA	46.750	3.750	2.623	0.538	0.562	0.167	3560	0.029
PG	44.917	3.583	2.403	0.548	0.554	0.000	3880	0.080
FG	47.000	3.583	2.336	0.567	0.510	0.083	3550	−0.097
TTZ	46.750	3.917	2.438	0.576	0.567	0.167	3570	−0.024
MAC	43.917	3.583	2.510	0.564	0.553	0.250	3580	0.058

*Na* number of alleles, *Ne* number of effective alleles, *Ho* observed heterozygosity, *He* expected heterozygosity, *Np* private alleles, *R* allelic richness, *Fis* inbreeding coefficient, *CAL* calçoene, *OIA* oiapoque, *PGR* Porto Grande, *FGO* Ferreira Gomes, *TTZ* Tartarugalzinho, *MAC* Macapá.

### Genetic diversity and differentiation

Table 1 shows the genetic diversity in the *Ae. aegypti* populations from the Amapá State. The average allelic diversity varied from 3.58 (MAC) to 3.91 (TTG), the average number of effective alleles (Ne) from 2.33 (FGO) to 2.62 (OIA), the number of private alleles per population (Np) from 0 (CAL and PGR) to 0.25 (MAC), and the number of allelic richness (R) from 3.56 (CAL) to 3.88 (TTZ). The genetic diversity of a mosquito population usually is positively related to the expected heterozygosity (He) and observed heterozygosity (Ho) values. Here the He varied from 0.510 to 0.567 and the Ho varied from 0.528 to 0.576 (Table 1).

Regarding the genetic differentiation as measured by the Fst, the values of the pairs ranged from 0.004 (PGR-TTZ) to 0.084 (OIA-PGR) (Table 2 and Supplementary Table S6). Except for the pairs OIA-FGO (0.818) and OIA-PGR (0.884), the number of migrants (Nm) values were greater than one, indicating a certain degree of gene flow between the *Ae. aegypti* populations along the BR-156 road. The pairs associated with OIA showed the highest Fst and, conversely, the lowest Nm values, which can be justified by the fact that Oiapoque is the city located in the extreme north of the BR-156 road. Interestingly, OIA-MAC showed the highest Nm value among all pairs with OIA, although Macapá and Oiapoque are the most distant cities. The AMOVA showed Fis and Fit values of 0.92 and 1.57 (both *p* < 0.001), respectively (Supplementary Table S7), indicating a moderate differentiation within the populations. At least 55% of the genetic distance among the populations may be attributed to isolation by distance (IBD), according to the Mantel test (0.549, *p* > 0.031).

**Table 2:** Genetic differentiation (Fst) and number of migrant (Nm) indexes of pairwise comparison of *Aedes aegypti* from Amapá State, Brazil.

Fst/Nm	CAL	OIA	PGR	FGO	TTZ	MAC
CAL	–	1.009	2.238	1.276	2.579	1.581
OIA	0.076	–	0.884	0.818	1.167	1.229
PGR	0.031	0.**084**	–	8.390	53.037	3.696
FGO	0.066	0.074	0.021	–	6.540	4.597
TTZ	0.034	0.067	**0.004**	0.016	–	3.569
MAC	0.040	0.050	0.026	0.031	0.019	–

*Bellow diagonal: Fst values; above diagonal: Nm values. Marked in bold are the highest and lowest Fst values. *CAL* calçoene, *OIA* oiapoque, *PGR* Porto Grande, *FGO* Ferreira Gomes, *TTZ* Tartarugalzinho, *MAC* macapá.

### Genetic structure

**Figure 2:**
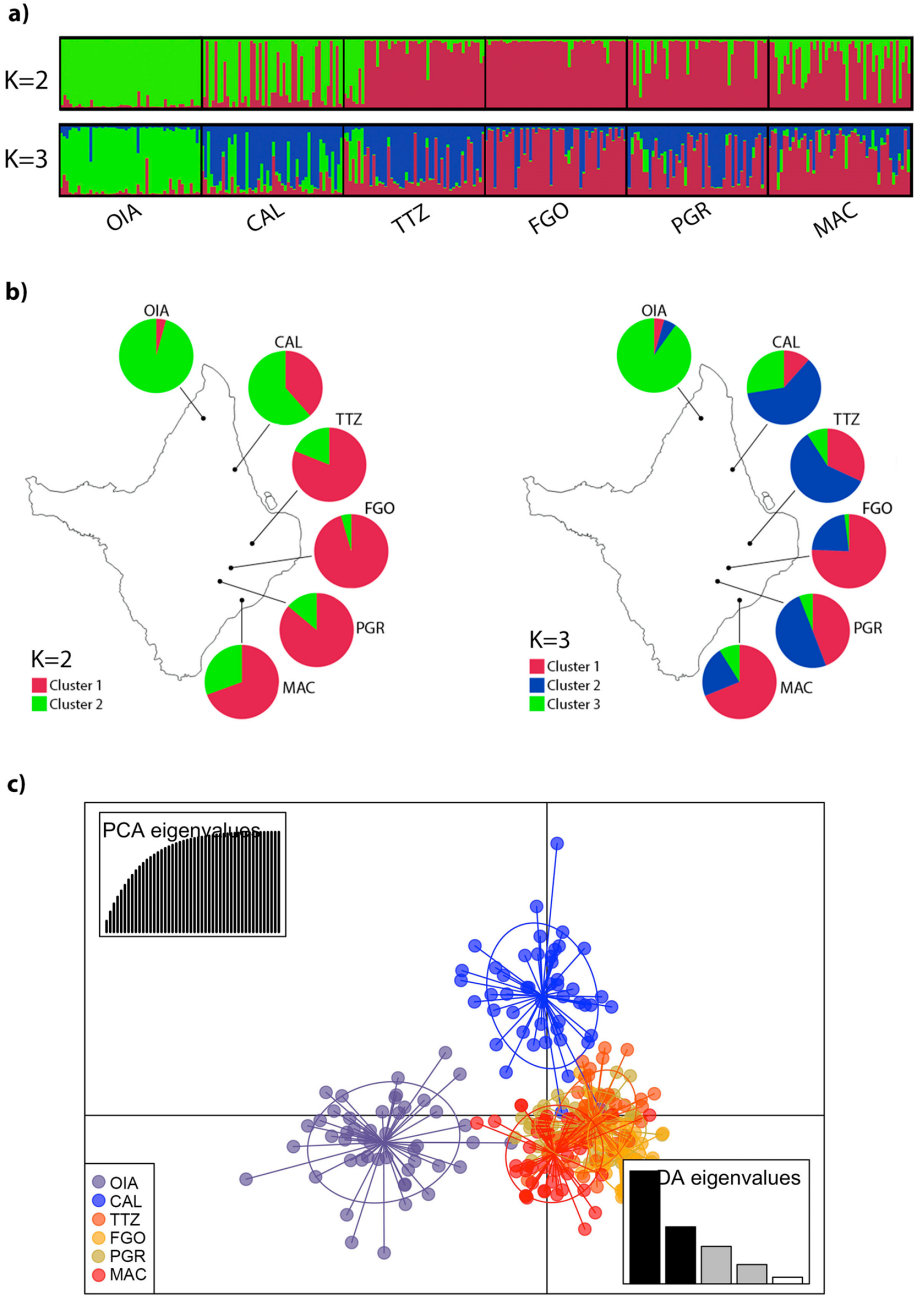
Genetic structure of *Aedes aegypti* populations from Amapá State, Brazil, based on 12 microsatellite markers. (**a**) Structure plot: Bayesian clustering analysis where each bar represents the inferred ancestry of each individual with two (K = 2) or three (K = 3) genetic clusters; (**b**) Pie charts of the global inferred ancestry value of each population with K = 2 and K = 3; (**c**) Discriminating analyses of principal components (DAPC) plot, with the dots and colors representing individuals and groups, respectively. Eigenvalues indicate the number of principal components that best explain the differences between individuals (4 components were indicated for the 6 populations).

The Bayesian analysis conducted on the mosquito populations from Amapá suggests that *Ae. aegypti* is most likely divided into two genetic clusters (K = 2) (Supplementary Figure S2). We designed the structure plot considering K = 2 and K = 3 (Fig. 2a). In both scenarios, TTZ, FGO, PGR and MAC are the most homogeneous populations. With K = 2, OIA and CAL seem more related; with K = 3, OIA stands out as a more isolated population. The pie charts with the cluster frequencies in each population over the map (Fig. 2b) facilitate observing the populations’ genetic structure along the BR-156 road. For example, with K = 2, we observed that the most homogeneous populations were OIA and FGO, with 95.7% of the genetic diversity assigned back to cluster 2 in OIA while 95.5% to cluster 1 in FGO. In addition, the DAPC analysis plot evidenced three groups, where each OIA and CAL formed isolated groups, and TTZ, FGO, PGR and MAC were all mixed in a third group (Fig. 2c). These genetic structure analyses complemented the genetic differentiation indexes, suggesting that OIA and CAL are more structured than the other populations. This trend can be explained by the likely higher gene flow among the mosquitoes from TTZ, FGO and PGR and the capital MAC compared to OIA, which is a distant and isolated city located in the border area with French Guiana.

### *Kdr* and genetic structure

**Figure 3:**
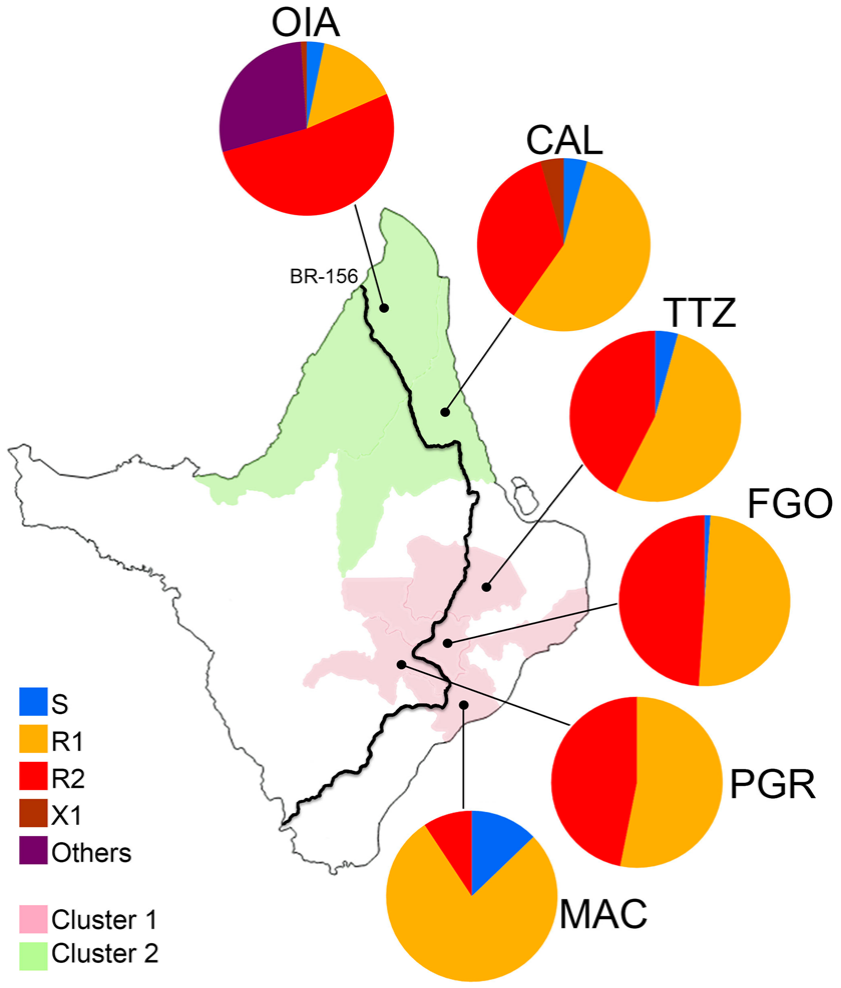
*Kdr* allelic frequencies and genetic clustering of *Aedes aegypti* populations from Amapá State, Brazil. *Kdr* allelic frequencies are indicated in pie charts for each respective locality. The localities are shaded according to their genetic clustering (K = 2, see Fig. 2). OIA: Oiapoque, CAL: Calçoene, TTZ: Tartarugalzinho, FGO: Ferreira Gomes, PGR: Porto Grande, MAC: Macapá. S: Wild-type *Na*_*V*_*S* (VVF), R1: *kdr Na*_*V*_*R1* (VVC), R2: *kdr Na*_*V*_*R2* (LIC), X1: *kdr Na*_*V*_*X1* (LVC), Others: alleles possibly composing the Y genotype: R2X2 (LIC/LVF) or X1X3 (LVC/LIF), see Fig. 1b.

Considering the genetic structure based on the SSR analyses into two clusters (K = 2), the populations from clusters 1 and 2 presented the *kdr* alleles *Na*_*V*_*R1* and *Na*_*V*_*R2*, whilst the additional *kdr* alleles were present only in the populations from cluster 2 (Fig. 3).

## Discussion

In this study, we investigated the frequency of *kdr* genotypes and the genetic structure of *Ae. aegypti* populations along a South-North transect of the Amapá State, starting from the capital city Macapá to Oiapoque, located on the border between Brazil and French Guiana. The Amapá State is particularly isolated from the other States and covered by > 90% by the Amazon Forest. Our study showed that the dengue vector *Ae. aegypti* is divided into two well-defined genetic clusters in this State, and the *kdr* allelic composition roughly followed this clustering. We also found evidence of new *kdr* genotype arrangements in at least two of the six mosquito populations analyzed. Better understanding of the genetic structure of *Ae. aegypti* populations is relevant not only to better understand the possible dispersion of resistance genes in this region but also to guide decision-making for vector control and insecticide resistance management.

Historically in Brazil, the organophosphate malathion replaced pyrethroid insecticides around 2006–2009^12^. However, malathion was never utilized in the Amapá State (operational constraints), and until recently, only pyrethroids were employed. The first cases of chikungunya in Brazil were recorded in 2014 in the cities of Oiapoque (Amapá State) and Feira de Santana (Bahia State), from two distinct events^25^. As a result, the use of pyrethroid insecticide (deltamethrin) was intensified in Oiapoque, which likely contributed to the increase in the selection pressure on resistance genes. Indeed, *Ae. aegypti* populations collected from Amapá State in 2014/2015 showed high resistance to deltamethrin with estimated RR_50_ (resistance ratio) of 46.4 and 143.9 in Macapá and Oiapoque, respectively^26^. Recently, Cielo has been used in ULV applications since 2019, and Spinosad was introduced in Amapá State in 2021 (*personal communication—Health Secretariat of Amapá State*). In French Guiana, deltamethrin is the only adulticide authorized in vector control campaigns since 2011^28^.

Here, we observed that all *Ae. aegypti* populations from Amapá presented high frequencies of *kdr* alleles, considering the three important SNPs in the voltage-gated sodium channel gene (*Na*_*V*_): V410L, V1016I and F1534C. Interestingly, we found two additional *kdr* genotypes composition or arrangement, LL + VI + CC in Calçoene and Oiapoque, and LL + VI + FC in Oiapoque. These distinct genotypes were probably present in previous surveys; however, they were not detected since the V410L had not been investigated in mosquitoes from Amapá^16^. The LL + VI + CC (R2X1) was previously observed in populations from Peru (Palomino, 2022—unpublished data), Colombia^29^, Mexico^30^, and USA^31^. The LL + VI + FC (Y) was also previously observed in populations from the countries mentioned above, and in distinct Brazilian regions, such as Natal, Salvador, Vitória and Brasília^16^. Regardless of the location, their frequencies were generally low: varying from 1.1% in the USA to 5.8% in Colombia (R2X1), and from 0.2% in Peru to 2.5% in the USA (Y). In Amapá the frequencies were 28.3% (Y) and 2.2% (R2X1) in Oiapoque, and 8.7% (R2X1) in Calçoene. Complementary studies conducted by our research team suggest that these genotypes may result from gene duplications, hence maintaining distinct copies of the *Na*_*V*_ gene in the same chromosome^32,18,16^. Their relationship with pyrethroid resistance requires further investigation.

We also showed that the *kdr Na*_*V*_*R2* allelic frequency remained very high in Oiapoque (67% in 2014/2015^26^, 68.5% in 2018^16^ and 52.2% in 2020) and it increased significantly in Calçoene (from 7.8% in 2018^16^ to 35.9% in 2020). While the *kdr Na*_*V*_*R2* was absent in Macapá in 2014/2015^26^, it has been found at low frequency (5.6%) in 2018^16^, and at 9.3% in 2020, as shown in this study. The homozygous R2R2 is still absent from Macapá but it has been reported in Calçoene for the first time. In the localities of Ferreira Gomes, Porto Grande and Tartarugalzinho *kdr Na*_*V*_*R2* varied from 42.4 to 48.9% and the R2R2 genotype ranged from 21.7 to 31.1%. It is worth noting that all localities along the BR-156 road had higher *kdr Na*_*V*_*R2* frequency than Macapá, which is the State capital and therefore highly connected to these localities. Considering the genetic cost of the *kdr Na*_*V*_*R2* under insecticide-free environment^18^, we assume that the insecticide pressure by pyrethroids might be lower in Macapá compared to other cities, hence prevailing the introduction and establishment of the *kdr Na*_*V*_*R2* in *Ae. aegypti* in the State capital.

In addition, the decrease of the wild-type *Na*_*V*_*S* allele in all populations is of great concern. In Macapá, the frequency of the S allele was 15.7% in 2014/2015^26^, 17.8% in 2018^16^ and 12.8% in 2020. We also showed that the wild-type allele *Na*_*V*_*S* is now absent in Porto Grande and less than 13% in other populations tested. It is worth noting that the *Na*_*V*_S allele was not detected in any *Ae. aegypti* population from the Amazonian region in the previous nationwide surveillance (2018)^26^.

Regarding the *kdr* alleles nucleotide composition, we showed that the sequences of R1R1 and R2R2 (homozygous) samples from Macapá and Oiapoque were similar to the ones previously described^17,33^. Evidence shows that at least two haplotypes emerged independently with the 1534C *kdr* mutation and one haplotype with the *kdr* mutation 1016I^17^. The 1534C is present in the herein called *kdr* alleles *Na*_*V*_*R1* (VVC) and *Na*_*V*_*R2* (LIC) and the 1016I in the *kdr Na*_*V*_*R2* in *Ae. aegypti* Brazilian populations^16^.

Overall, we suspect that the high resistance to pyrethroids in Oiapoque is associated with a higher frequency of *kdr Na*_*V*_*R2* and the occurrence of rare *kdr* genotypes, as well as to the higher expression of detoxifying genes. Indeed, *Ae. aegypti* populations collected in the transborder city of Saint Georges de Oiapoque in French Guiana showed marked amplification of several *CYP6s* and *CYP9Js* playing a role in pyrethroid resistance^34^. We then cannot discard the possibility that these genes may be present in the Oiapoque population. Indeed Oiapoque (AP-Brazil) and Saint-Georges (French Guiana) are separated by a river which probably does not avoid the intense gene flow of *Ae. aegypti* between both sides, facilitated by the transportation of people and goods, which directly impacts the genetic structure of the mosquito^24^. Further work is needed to assess the detoxification pathway and genomic changes underlying the resistance mechanism of *Ae. aegypti* in the transborder area between Brazil and France that exhibits extremely high levels of resistance to all public health insecticides.

Finally, we analyzed the genetic structure and gene flow between *Ae. aegypti* from Amapá using 12 microsatellites markers. We showed that the Fst values among the six populations of *Ae. aegypti* were higher in the pairs with Oiapoque, which was isolated in a single genetic group in the Bayesian analysis (k = 2) and clearly isolated in the DAPC. Calçoene, the closest city to Oiapoque, was also represented in an isolated group in the DAPC. The isolation by distance (IBD), confirmed by the Mantel test, and the dense Amazon Forest remain the most likely explanation for the genetic structure and *kdr* frequency differences among the populations alongside the BR-156 Road. Interestingly, Oiapoque presented the lowest Fst and the highest Nm with Macapá, that are yet distant from 580 km. This can be explained by the fact that Macapá is the State capital, and the flow of people and goods between those cities is very intense, hence facilitating the passive transportation of *Ae. aegypti*.

Our findings align with other studies that support the hypothesis attributing the long-distance dispersal of *Ae. aegypti* to human activities, particularly vehicular traffic on highways^35,36^, as well as boat^37,38^ and airplane^39^ transportation. A prior study identified the spread of resistance and *kdr* mutations within *Ae. aegypti* populations along a major road in central Brazil. The movement of people and goods between the state capital and other cities was claimed as the factor facilitating the dispersal^35^. A mitochondrial DNA based study of *Ae. aegypti* populations from Argentina suggested that this mosquito passively commuted the long distance of approximately 60 km in a year^36^. In the Peruvian Amazon, the *Ae. aegypti* gene flow was higher between localities with heavy boat traffic compared to those with less intense movement^37^, similarly to our findings, however with the transportation in the road encased within a dense forest.

Based on the use of microsatellite markers, some studies concluded that the reinfestation of *Ae. aegypti* populations in Brazil after the eradication programme of the 1970s may have come from at least two distinct genetically differentiated groups, i.e. one from Venezuela to northern Brazil, and one from the Caribbean populations to the southeast of Brazil^40,41^. From this perspective, the Amapá State should have been invaded by *Ae. aegypti* from the two genetically distinct groups: in the north (Oaipoque and Calçoene) by the ‘Caribbean group’ through French Guiana, and in the south (Macapá, Ferreira Gomes, Porto Grande and Tartarugalzinho) by the ‘Venezuelan group’ through other Amazonian Brazilian locality, probably Pará State.

In this study, we used two types of molecular genotyping, *kdr* and microsatellite markers, to address distinct questions. The *kdr* SNPs were genotyped to obtain their genotypic frequencies in the populations. As *kdr* is under strong selection pressure, it is not an accurate marker to infer the population genetic structure, which was achieved by neutral microsatellite genotyping. By combining these two analyses, we observed that *kdr* alleles are spreading, likely by the *Ae. aegypti* ability to disperse itself and its eggs passively, however, without disrupting the overall genetic structure of the isolated populations.

Microsatellites and *kdr* genotyping analyses evidenced genetic differences among *Ae. aegypti* populations relatively isolated by a dense forest, connected by an only principal road. We found that rare *kdr* genotypes are increasing in frequency in some regions of Amapá State and this relationship with pyrethroid resistance deserves further investigation. More additional work is also needed to better understand the environmental and landscape determinants involved in the evolution and spatial distribution of insecticide resistance including *kdr* mutations in the dengue vector *Aedes aegypti* in Amapá.

## Methods

### *Ae. aegypti* collections

**Figure 4:**
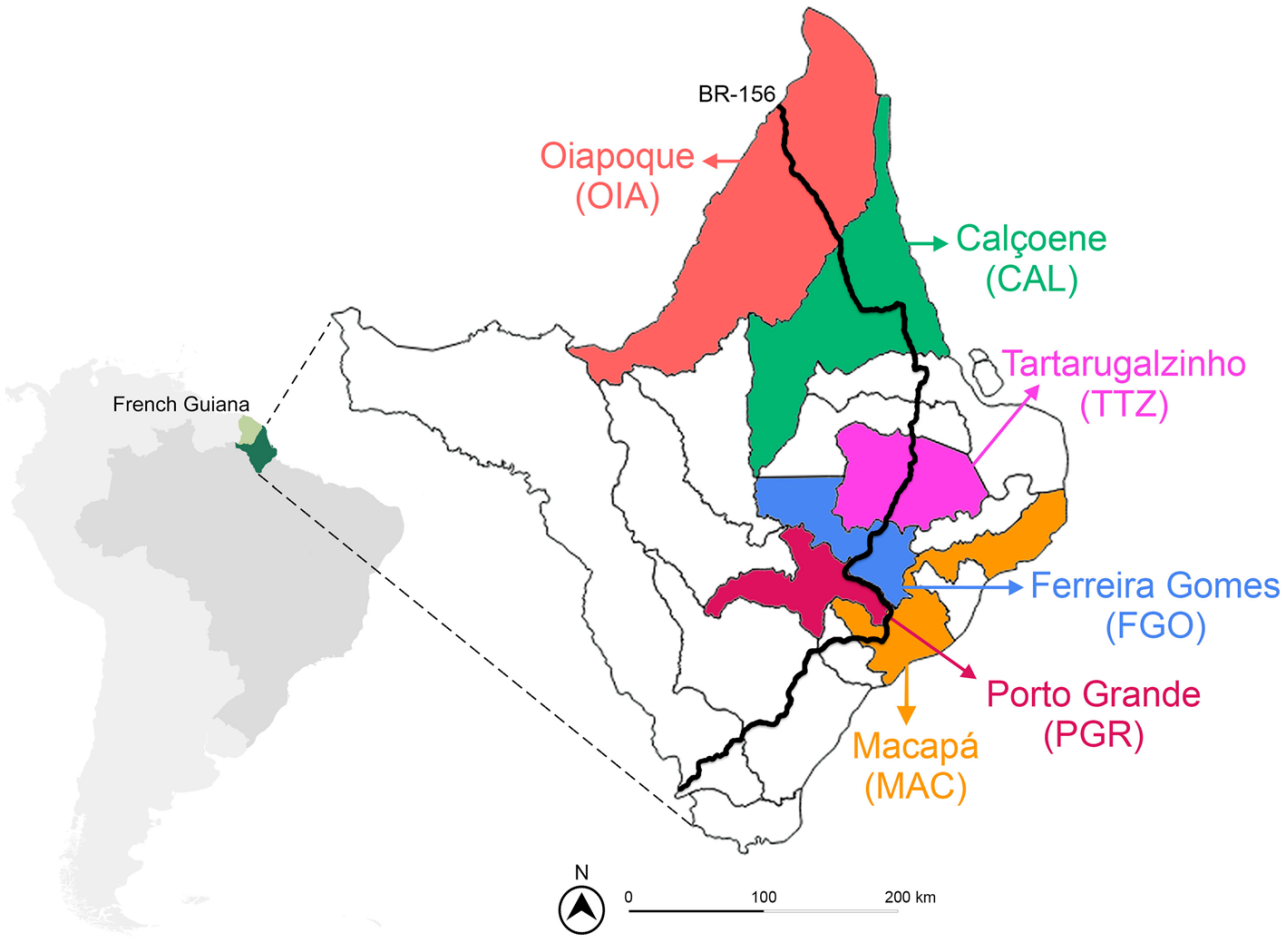
Map showing the location of Amapá State, Brazil, and French Guiana, France. The delimitations of Amapá cities are also shown including the cities where *Aedes aegypti* were collected (in colour). The BR-156 road is indicated by a black line.

The field team of *Laboratório de Entomologia Médica do Instituto de Pesquisas Científicas e Tecnológicas do Estado do* Amapá (IEPA) installed 50 eggtraps in each of the six Amapá cities: Oiapoque (OIA) (03°49′53'' N, 51°50′07'' W), Calçoene (CAL) (02°29′53'' N, 50°56′59'' W), Tartarugalzinho (TTZ) (01°30′21” N, 50°54′41'' W), Ferreira Gomes (FGO) (00º51′14 “ N, 51º11′39″ W), Porto Grande (PGR) (00°42′16'' N, 51°24′35'' W) and Macapá (MAC) (00°02′04″ N, 51°03′60″ W) (Fig. 4), following the methodology developed by the Ministry of Health^13^. Collections were made in August (OIA, CAL, TTZ, FGO, PGR) and October/2020 (MAC). The eggtraps in CAL, FGO, PGR and TTZ were randomly distributed in the whole city. In MAC and OIA, the eggtraps were preferentially placed on the sidelines of BR-156 road. The eggs were stimulated to hatch in IEPA laboratory, and the resulting adults (F0) were packed in silica gel and shipped to *Laboratório de Biologia, Controle e Vigilância de Insetos Vetores* (LBCVIV) at *Instituto Oswaldo Cruz* (IOC/Fiocruz) for further analysis.

### *Kdr* genotyping

We extracted DNA from male mosquitoes from the F0 generation (from egg collection), individually titrated with TNES buffer, as described elsewhere^42^. The DNA of each sample was quantified in a Nanodrop One C spectrophotometer (Thermo Scientific) and aliquoted at 20 ng/µL in ultra-pure water. About 45 individuals from each population were genotyped for the *kdr* SNPs V410L, V1016I and F1543C through real-time TaqMan qPCR approach, essentially as described elsewhere^16^ (See Supplementary Table S8 for primers and probes sequences). The genotyping callings of each SNP were resolved by the online software Genotype Analysis Module v3.9 (Thermo Fisher), setting the endpoint to CT 40. Allelic and genotypic frequency charts were performed on GraphPad Prism v9.2.0 (www.graphpad.com). We considered the three SNPs (410 + 1016 + 1534) to determine the genotype of each sample^16,20^.

### ***Na***_***V***_ sequencing

We amplified and sequenced fragments corresponding to the IS6, IIS6, and IIIS6 *Na*_*V*_ segments of elected samples to determine the respective *kdr* haplotypes circulating in *Ae. aegypti* populations from Amapá. We selected nine samples genotyped as homozygous to *kdr Na*_*V*_*R1* or *kdr Na*_*V*_*R2* alleles (see Results) from MAC and OIA. For the PCR amplification, we used the kit Phusion High-Fidelity PCR (New England, Biolabs), containing Phusion Buffer 1X, DMSO 3%, the respective primers pair^30,43,44^ (Supplementary Table S9), 0.5 µm each and ultra-pure water q.s. 25 µL. The thermal-cycle conditions were 98 °C/30″ in the first step, followed by 35 cycles at 98 °C/10″ for denaturation, 60 °C (IS6), or 57 °C (IIS6), or 61 °C (IIS6)/15″ for primers annealing, and 72 °C/30″ for the Polymerase amplification, followed by a final extension step at 72 °C/7’. The amplicons were purified with the Qiaquick PCR Purification kit (Qiagen), according to the conditions provided by the manufacturer, and subjected to the sequencing reaction with the Bigdye Terminator V3.1 kit (Invitrogen, Thermo Fisher) using 1 µL of the purified amplicon and 1 µM of one of the respective primers. The sequencing reaction products of both strands of each sample were sent to the Fiocruz DNA Sequencing Facility (an ABI 3730 equipment). The sequences were analyzed using Geneious v9.1.8^45^.

### Microsatellites genotyping

The same DNA samples used for *kdr* genotyping were also genotyped for 12 well-characterized and polymorphic microsatellite loci that are widely used in studies of *Ae. aegypti* populations from the American continent: AC1, AC2, AC4, AC5, AG1, AG2, AG5, CT2, A1, A9, B2 and B3 (Supplementary Table S10)^46,47^. We performed the reactions with the Type-It PCR kit (Qiagen), according to a protocol standardized by Brown et al.^46^, using multiplex primers, originally proposed by Schuelke^48^, using an M13 tail at the primers 5' end and marked with FAM or HEX fluorescence (Supplementary Table S10). For each reaction, we used the Type-It Multiplex PCR Master Mix (Qiagen) 1x, each forward primer at 0.025 μM, each reverse primer at 0.25 μM, each probe at 0.5 μM, 1 µL of DNA (20 ng) and ultra-pure water q.s. 10 µl. The thermocycling conditions were 94 °C/10’ followed by 35 cycles of 94 °C/30″, 54 °C/30″ and 72 °C/30″, followed by the final step of 72 °C/5’. The product of each PCR was diluted 1:10, and 1 µL of the product was used for genotyping. Each amplicon received 0.5 μM the dye size standard GeneScan 500 LIZ (Applied Biosystems) and was submitted to the Genotyping/ Fragment Analysis Facility at Fiocruz (equipment 3130xl Genetic Analyser, Applied Biosystems).

### Data analysis

We used the software *Geneious* 9.1.8^45^ with the *plug-in* developed for ABI fragment analyses (*Geneious Microsatellite Plugin*) to obtain the genotype callings of all microsatellite loci, which were exported in a .csv file. We tested the loci for Hardy–Weinberg equilibrium (HWE) and linkage disequilibrium in the Genepop v4.7.5 software, with significance levels adjusted by Bonferroni correction^49,50^. The presence of null alleles in each locus was verified with Microchecker Software v2.2.3^51^. The genetic diversity parameters: average number of different alleles (Na), number of effective alleles (Ne), private allelic richness (Np), expected heterozygosity (He), observed heterozygosity (Ho), endogamy coefficients (Fis) and the number of migrants (Nm) for each population were estimated using the software Genetic Analysis in Excel (Genalex) v.6.503^52^. This same software was used for analysis of molecular variance (AMOVA). The allelic richness (R) was calculated with the HP-Rare v1.1 software^53^. We estimated the magnitude of genetic differentiation with paired Fst values using Arlequin v3.5.2^54^ and Freena^55^, respectively, both with 10,000 permutations. To test the assumption of isolation by distance (IBD), we run the Mantel test, with Arlequin v3.5.2 with 10,000 permutations^54^, correlating genetic and geographic data (geographical distance per km × Fst).

We also used the Structure v2.3.4 software^56^ to assess the population's genetic structuring based on the number of genetic clusters (K). The best K value was obtained based on ten independent runs with 500,000 Monte Carlo chains (MCMC) iterations, excluding 20% of the initial chains (burn-in). The output data was analyzed in the Structure Harvester 2.3 software^57^ to determine the best number of genetic clusters (K) based on an analysis of the ΔK chart^58^. Finally, we submitted the output files to Clumpp v1.1.2^59^ and Distruct V1.1^60^ software to plot the genetic structure of the evaluated populations. A multivariate statistical analysis (discriminant analysis of principal components—DAPC) was performed with the Adegenet package^61,62^ in the R platform^63^.

### Supplementary Information

Supplementary Information 1.

Supplementary Information 2.

Supplementary Information 3.

Supplementary Information 4.

## Data Availability

The data presented in this study are openly available in FigShare at https://figshare.com/s/6cabba153e11a4b8e9a1.
