# Supplementary figures and images for: Genetic structure and kdr mutations in Aedes aegypti populations along a road crossing the Amazon Forest in Amapá State, Brazil
Source: Sci Rep. 2023 Oct 11;13:17167. doi: 10.1038/s41598-023-44430-x (PMC10567682; doi:10.1038/s41598-023-44430-x)

**OIA**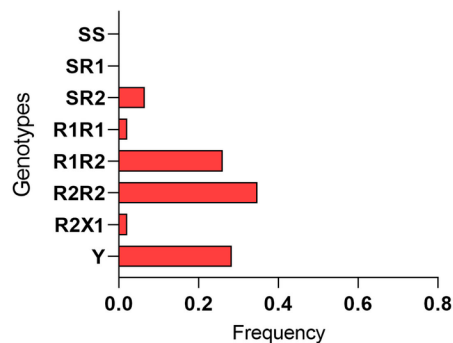**CAL**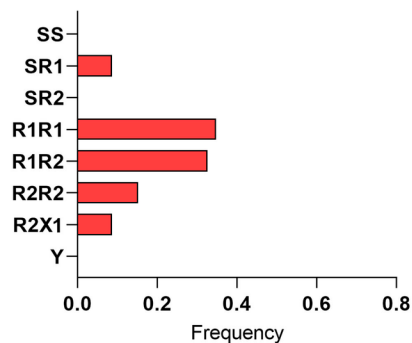**TTZ**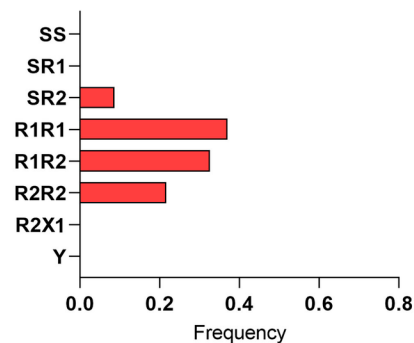**FGO**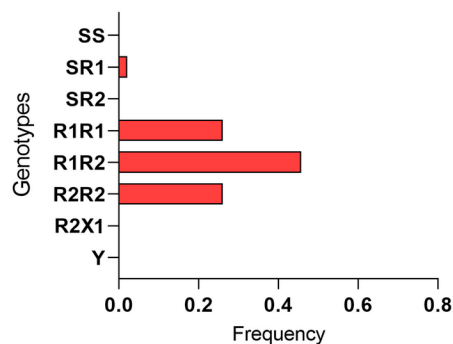**PGR**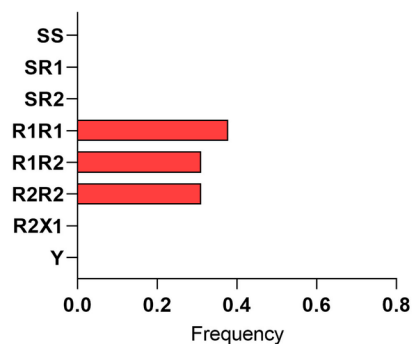**MAC**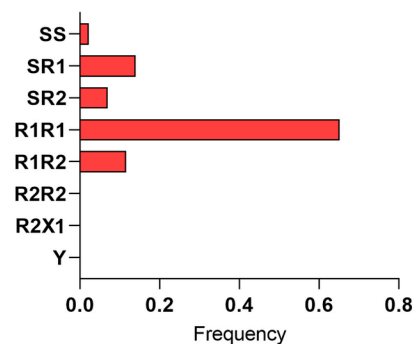

Supplement: Supplementary file 1 — Supplementary Information 1. [file 41598_2023_44430_MOESM1_ESM.pdf]

$$\text{DeltaK} = \text{mean}(|L''(K)|) / \text{sd}(L(K))$$

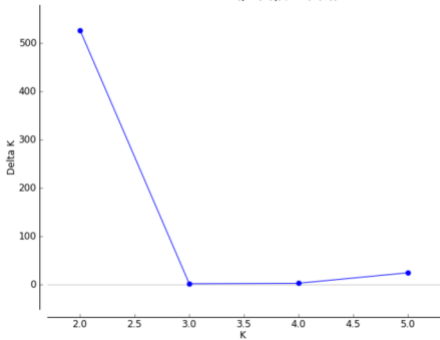

Supplement: Supplementary file 2 — Supplementary Information 2. [file 41598_2023_44430_MOESM2_ESM.pdf]
