# Supplementary material for: Genetic structure and kdr mutations in Aedes aegypti populations along a road crossing the Amazon Forest in Amapá State, Brazil
Source: Sci Rep. 2023 Oct 11;13:17167. doi: 10.1038/s41598-023-44430-x (PMC10567682; doi:10.1038/s41598-023-44430-x)
Supplement: Supplementary file 4 — Supplementary Information 4. [file 41598_2023_44430_MOESM4_ESM.pdf]

Supplementary Materials (legends)

Supplementary Figure S1. Frequency of *kdr* genotypes for each *Aedes aegypti* population in the Amapá State, Brazil.

Supplementary Figure S2. Evanno plot derived from Structure Harvester to detect the number of genetic clusters.

Supplementary Table S1. Frequencies of *kdr* genotypes in *Aedes aegypti* populations from the Amapá State, Brazil, considering the NaV SNPs V410L, V1016I, F1534C.

Supplementary Table S2. Frequencies of *kdr* alleles in *Aedes aegypti* populations from the Amapá State, Brazil, considering the NaV SNPs V410L, V1016I, F1534C.

Supplementary Table S3. Allele frequencies of the 12 microsatellite loci in *Aedes aegypti* populations from the Amapá State, Brazil.

Supplementary Table S4. Analysis of the Genetic Diversity of *Aedes aegypti* populations from the Amapá State, Brazil, using 12 microsatellite loci.

Supplementary Table S5. Analysis of linkage disequilibrium of *Aedes aegypti* populations from the Amapá State, Brazil.

Supplementary Table S6. Genetic differentiation (Fst) index of pairwise comparison of *Aedes aegypti* from Amapá State, Brazil.

Supplementary Table S7. Analysis of molecular variance (AMOVA) of *Aedes aegypti* from Amapá State, Brazil.

Supplementary Table S8. Primer and probe sequences for the SNPs V410L, V1016I and F1534C *kdr* in *Aedes aegypti* populations from the Amapá State, Brazil.

Supplementary Table S9. Primers used to amplify the IS6, IIS6 and IIIS6 fragments of the voltage-gated sodium channel gene (*NaV*) from populations of *Aedes aegypti* from Amapá, Brazil.

Supplementary Table S10. Primers for microsatellite genotyping in *Aedes aegypti* populations from the Amapá State, Brazil.
